# Colorectal Cancer Patient‐Derived 2D and 3D Models Efficiently Recapitulate Inter‐ and Intratumoral Heterogeneity

**DOI:** 10.1002/advs.202201539

**Published:** 2022-06-02

**Authors:** Yuanyuan Zhao, Bing Zhang, Yiming Ma, Fuqiang Zhao, Jianan Chen, Bingzhi Wang, Hua Jin, Fulai Zhou, Jiawei Guan, Qian Zhao, Hongying Wang, Qian Liu, Fangqing Zhao, Xia Wang

**Affiliations:** ^1^ School of Pharmaceutical Sciences Tsinghua University Beijing 100084 China; ^2^ Beijing Institutes of Life Science Chinese Academy of Sciences University of Chinese Academy of Sciences Beijing 100101 China; ^3^ State Key Laboratory of Molecular Oncology National Cancer Center/National Clinical Research Center for Cancer/Cancer Hospital Chinese Academy of Medical Sciences and Peking Union Medical College Beijing 100021 China; ^4^ Department of Colorectal Surgery National Cancer Center/National Clinical Research Center for Cancer/Cancer Hospital Chinese Academy of Medical Sciences and Peking Union Medical College Beijing 100021 China; ^5^ Department of Pathology National Cancer Center/National Clinical Research Center for Cancer/Cancer Hospital Chinese Academy of Medical Sciences and Peking Union Medical College Beijing 100021 China; ^6^ Key Laboratory of Systems Biology Hangzhou Institute for Advanced Study University of Chinese Academy of Sciences Hangzhou 310024 China; ^7^ Center for Excellence in Animal Evolution and Genetics Chinese Academy of Sciences Kunming 650223 China

**Keywords:** colorectal cancer, organoids, preclinical model, tumor heterogeneity

## Abstract

Pre‐existing drug resistance and tumorigenicity of cancer cells are highly correlated with therapeutic failure and tumor growth. However, current cancer models are limited in their application to the study of intratumor functional heterogeneity in personalized oncology. Here, an innovative two‐dimensional (2D) and three‐dimensional (3D) model for patient‐derived cancer cells (PDCCs) and air–liquid interface (ALI) organotypic culture is established from colorectal cancer (CRC). The PDCCs recapitulate the genomic landscape of their parental tumors with high efficiency, high proliferation rate, and long‐term stability, while corresponding ALI organotypic cultures retain histological architecture of their original tumors. Interestingly, both 2D and 3D models maintain the transcriptomic profile of the corresponding primary tumors and display the same trend in response to 5‐Fluoruracil, regardless of their difference in gene expression profiles. Furthermore, single‐cell‐derived clones() are efficiently established and pre‐existing drug‐resistant clones and highly tumorigenic clones within individual CRC tumors are identified. It is found that tumorigenic cancer cells do not necessarily possess the stem cells characteristics in gene expression. This study provides valuable platform and resource for exploring the molecular mechanisms underlying the pre‐existing drug resistance and tumorigenicity in cancer cells, as well as for developing therapeutic targets specifically for pre‐existing drug‐resistant or highly tumorigenic clones.

## Graphical abstract

Colorectal cancer 2D patient‐derived cancer cells (PDCCs) and 3D air–liquid interface (ALI) organoids are established in this paper. This 2D and 3D model recapitulates histological, genomic and transcriptomic characteristics of parental tumors. Furthermore, the PDCCs show inter‐ and intratumoral heterogeneity of drug sensitivities and tumorigenicity. This 2D and 3D model can be applied to preclinical research more effectively and systematically.

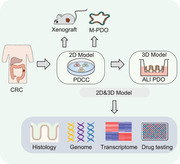

## 1 Introduction

Tumor heterogeneity is a major challenge and puzzle in oncology research and clinical practice.^[^ ^1^ ^]^ Intratumor heterogeneity is now recognized as a critical factor associated with therapeutic failure, tumor growth, and drug resistance.^[^ ^2^, ^3^ ^]^ Hence, the measurement and modeling inter‐ and intratumoral heterogeneity has become a key clinical issue. Currently, intratumor heterogeneity can be revealed by multiregional sequencing,^[^ ^4^ ^]^ single‐cell sequencing,^[^ ^5^ ^]^ imaging,^[^ ^6^ ^]^ etc. However, current two‐dimensional (2D) and three‐dimensional (3D) cancer models remain limited in their application to study intratumor functional heterogeneity in personalized oncology. Notably, Clevers’ group implemented the first leap forward using single‐cell derived organoids, but only for drug response.^[^ ^7^ ^]^ Intratumor heterogeneity studies of other important clinically relevant functions, such as tumorigenicity, remain unexplored.

There is a growing consensus that improvement of preclinical models could facilitate the discovery and development more effective therapeutic regimens for patients.^[^ ^1^, ^8^ ^]^ Traditional 2D cancer cell lines are the most commonly used cancer models.^[^ ^9^ ^]^ The advantage of 2D cultures is easy of manipulation and observation. However, establishing a new 2D cancer cell line is an extremely inefficient, challenging, and time‐consuming process. Only extremely rare clones have adapted to selection and culture conditions, so they cannot reproduce the complete diversity of primary tumors, not suitable for studying tumor heterogeneity.^[^ ^10^ ^]^ The more advanced 3D patient‐derived organoids (PDOs) can better recapitulate the physiological features of their parental tumors and capture the inter‐ and intraheterogeneity.^[^ ^7^, ^11^ ^]^ However, organoids are heterogeneous in terms of size, shape, and viability even within the same culture, and the relatively rigid Matrigel may limit drug penetration, all of which impedes drug screens.^[^ ^12^ ^]^ Therefore, the obvious demerits of current 2D and 3D models greatly limit their application in the modern era of individualized oncology study. And the combination of these 2D cancer cell lines and 3D organoids will substantially improve patient‐derived cancer models to meet the needs of personalized treatment.

The first challenge is to accomplish the high efficiency of generating patient‐derived cancer cell lines. Until 2015, by optimizing the 3T3 coculture system,^[^ ^13^ ^]^ we previously developed a long‐term and efficient culture of 2D human intestinal stem cell (ISC) clones,^[^ ^14^ ^]^ as well as air–liquid interface (ALI) cultures of 3D intestinal epithelium.^[^ ^15^ ^]^ The surface exposure and basal side nutrition supply characteristics make the ALI system very suitable for investigating the interaction between exogenous compounds and cells. In view of these advantages, we need to further explore and verify whether this powerful 2D and 3D model can be used as a personalized cancer model for colorectal cancer.

Here, we successfully established CRC patient‐derived cancer cells (PDCCs) and ALI organotypic cultures with high efficiency and long‐term stability. This 2D PDCC lines showed extremely high proliferation rate, while this 3D ALI organotypic cultures showed physiologically differentiated tissue architecture with open apical polarity. We also successfully isolated and expanded single‐cell‐derived clones (SC‐PDCCs) from individual tumors to study the intratumoral heterogeneity. This combined 2D and 3D culture system (2D and 3D model) faithfully recapitulates the characteristics of the primary tumors and captures the inter‐ and intratumoral heterogeneity of drug response and tumorigenicity.

## 2 Results

### 2.1 Establishment of a 2D PDCC Model for CRC

**Figure 1:**
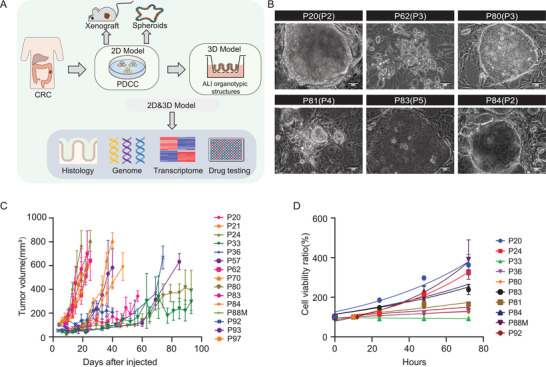
Establishment of CRC patient‐derived 2D PDCCs. A) Overview of experimental design. 2D PDCC lines were derived from CRC tissues; 3D ALI organotypic cultures, spheroids, and xenografts were generated from PDCC lines. The combined 2D and 3D culture system is called “a 2D and 3D model,” which can reflect patient‐specific characteristics at multiple levels, including histology, genome, transcriptome, and drug response. B) Representative bright field microscopy images of PDCC colonies. Passage numbers were marked in brackets. Scale bar: 50 µm. C) Tumor growth curves of 15 PDCC lines. 10^6^ cells were subcutaneously injected to five to six BALB/c nude mice for each PDCC line. Each line indicates one PDCC line, error bars indicate SD. D) Growth behavior of PDCCs in vitro. Quantification of the ratio of the number of cells counted per well at each time point to the number of cells at time point 0 of the same PDCC line (*n* = 10). Results are expressed as mean ± SD of three independent cultures. Figure 1A created with BioRender.com.

Although patient‐derived colorectal cancer organoids have previously been well established,^[^ ^11^, ^16^ ^]^ efficient primary culture of 2D colorectal cancer cell lines has remained a challenge for decades. We previously established an efficient culture method for 2D human ISCs with the irradiated 3T3‐J2 fibroblasts.^[^ ^15^ ^]^ Here, we optimized that method to establish a 2D PDCC model (see Experimental Section). In this, surgically resected tumor tissues and adjacent normal biopsies were obtained from consenting CRC patients (Table S1, Supporting Information). Each individual specimen was split into two parts, one for histological analysis, and the other for PDCC derivation, genomic and transcriptomic analyses (**Figure** **1**A). We successfully generated a CRC biobank with a total of 30 PDCCs lines from 39 tumor tissues (77%), and 22 normal colonic stem cells (NSCs) lines from 22 normal tissues (100%). In the future, sample quality assessment (by excluding samples with low cellularity or massive necrosis) and avoiding contamination can further increase the success rate of PDCC production. PDCCs formed individual colonies on the 3T3‐J2 feeder layer (Figure 1B). Of note, immune, vessel, and stroma cells did not form colonies on the 3T3‐J2 feeder layer. The number of cancerous colonies directly generated from primary tissues varied greatly between patients, ranging from less than ten to thousands. The size of the colonies also varied greatly between patients, ranging from 87 to 467 µm (Figure S1A, Supporting Information). The colony morphology of PDCCs presented intertumor heterogeneity (Figure 1B). Some cancerous colonies were tightly aggregated with very small cancer cells, some were loose with big cancer cells, and some were of an intermediate state. In general, the colony phenotypes of PDCCs were more irregular and variable, unlike those of NSCs. Like NSCs, PDCCs could be efficiently recovered (100% success rate, *n* > 22) after long‐term cryopreservation.

The growth rate of PDCCs showed intertumor heterogeneity. The stable passaging ratios of PDCCs ranged from 1:4–1:7 (≈39%) every 12 days to 1:10–1:200 (≈61%) every 7 days, while the passaging ratio of NSCs (1:20) was consistent between patients (Table S1, Supporting Information). Moreover, we evaluated the growth rate of PDCC‐derived xenografts in mice by subcutaneous transplantation of PDCCs into immunodeficient mice (BALB/c Nude). A total of 15 PDCC lines from 19 patients were successfully transplanted, with five to six xenografts per PDCC sample. All xenografts were examined at 3–12 weeks, and the success rate was 79%. The volume of xenografts with high growth rate reached 500 mm^3^ within 20–50 days, whereas xenografts with relatively low growth rate remained below 500 mm^3^ beyond 80–90 days (Figure 1C; Table S2, Supporting Information). By comparison, we found that 2D PDCCs displayed a highly concordant growth rate both in vivo and in vitro (Figure 1D). PDCCs have been continuously propagated for greater than 25 passages, without change in growth rate and morphology.

Taken together, this 2D culture is a very convenient model with advantages of high efficiency, rapid proliferation, and time saving.

### 2.2 2D PDCC‐Derived 3D ALI Organotypic Cultures and Xenografts Maintain the Histological Features of Original CRC Tissues

**Figure 2:**
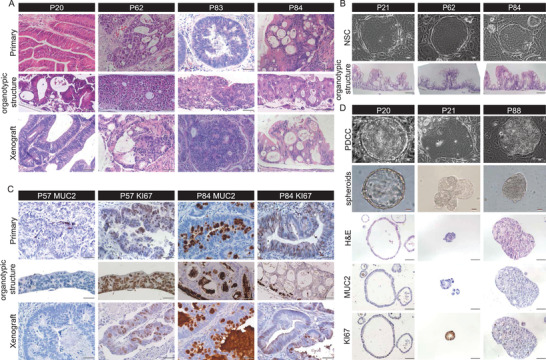
2D PDCCs‐derived 3D ALI organotypic cultures and xenografts retain histologic features of their parental tumors. A) Representative H&E staining images of 3D ALI organotypic cultures, xenografts, and matched primary tumors. Scale bar: 50 µm. B) Brightfield images of NSCs derived from noncancer tissues in three patients. Scale bar: 50 µm. H&E staining of corresponding ALI organotypic cultures. Scale bar: 50 µm. C) Concordant expression of MUC2 and KI67 in primary tumors and their derived ALI organotypic cultures and xenografts. Scale bar: 50 µm. D) Brightfield, histological and immunohistochemically images of spheroids derived from PDCCs. Scale bars: 50 µm.

We have demonstrated that ALI cultures generated from 2D ISCs faithfully recapitulate intestinal epithelium properties using a transwell‐based system.^[^ ^15^ ^]^ So, we are wondering whether the ALI cultures can simulate the morphological characteristics of primary tumors in vitro. We planted PDCC after 4–5 passage on ALI system. In the ALI condition, PDCCs formed 3D structures in 7–14 days and these ALI organotypic cultures could be maintained 15–30 days in vitro with a 100% success rate (20/20; **Figure** **2**A; Figure S1B, Supporting Information).

To assess whether ALI organotypic cultures and xenografts retain the morphological and histological features of their corresponding parental tumors, we performed H&E staining and immunohistochemical analyses. We observed that both ALI organotypic cultures and xenografts derived from pooled PDCCs revealed histological heterogeneity among patients, but still resembled their corresponding parental tumors (Figure 2A; Figure S1B, Supporting Information). Unlike the well‐organized and polarized epithelium displayed by normal ALI cultures, CRC ALI organotypic cultures displayed multiple different histological characteristics, including polarization, nonpolarization, poor organization, and vesicle‐like structures (Figure 2A,B). These multiple tumor characteristics were also observed in xenografts, whereas NSCs did not form xenograft tumors in mice (Figure 2A; Figure S1B, Supporting Information). Furthermore, MUC2 staining patterns showed abundant or deficient mucus proteins in both ALI organotypic cultures and xenografts, which were consistent with their corresponding parental tumors (Figure 2C; Figure S1C, Supporting Information). We also found varying amounts of Ki67‐positive tumor cells in the primary tumors, which were faithfully preserved by their derived ALI organotypic cultures and xenografts (Figure 2C; Figure S1C, Supporting Information). Particularly, histological features of ALI organotypic cultures (*n* = 8) and xenografts (*n* = 2) remained unchanged after long‐term culture of SC‐PDCCs for 10 passages and 20 passages, respectively (Figure S1D, Supporting Information). We then demonstrated that 2D PDCC can efficiently convert to spheroids under classical PDO culture condition (7/7, success rate 100%; Figure 2D; Table S1, Supporting Information).^[^ ^11c^ ^]^ The passaging ratios of both ALI organotypic cultures and spheroids ranged from 1:3 to 1:4, which was dramatically lower than that of 2D PDCCs (mostly 1:10 to 1:200). The MUC2 and Ki67 staining of spheroids were also consistent with the ALI organotypic cultures derived from 2D PDCCs (Figure 2D).

In summary, 2D PDCCs retain the exceptional potential of preserving histological characteristics, and when converted to 3D ALI organotypic cultures or spheroids in vitro and xenografts in vivo, they exhibit histological characteristics similar to those of original tumor tissues.

### 2.3 Genomic Characterization of PDCCs

**Figure 3:**
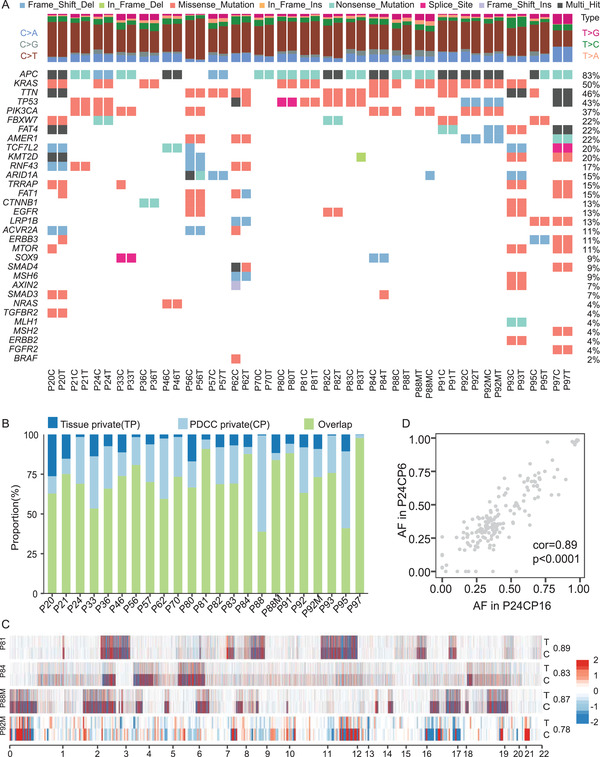
PDCCs maintain genomic landscape of corresponding tumors. A) Somatic mutations found in CRC related genes are present and conserved between paired PDCCs and parental tumor (T, tissues; C, PDCCs). Mutation type is indicated in the legend. The allele frequencies of these genes are listed in Figure S3E of the Supporting Information. *n* = 23, different patient samples. B) Bar plot of the concordance analysis of all SNVs detected in the PDCC lines and corresponding tissues. C) Heatmap visualization of inferred log R ratio (LRR) of CNAs comparing PDCCs and tissues. Numbers on the right side indicate the Pearson correlation coefficient for each pair of samples. Red denotes copy number gains, blue denotes copy number loss. D) Scatter plot comparing the somatic mutant fraction of SNVs for short‐time (passage 6) and long‐time culture (passage 16) of P24. Cor = 0.89; *p* < 0.0001. Correlations and *p* values are calculated by Pearson's correlation method.

To assess whether 2D PDCCs retain the genomic alterations of their corresponding parental tumors, we performed whole exome sequencing analysis. In total, we sequenced matched samples from 23 patients, including primary tumors and their derived PDCC lines, as well as the corresponding adjacent normal biopsies for references. We first measured the tumor purity in both PDCCs and their parental tumors. Across all samples, the percentage of cancer cells in PDCCs (72.6% ± 22.3%) was significantly higher than that of parental tumors (43.9% ± 20.3%; Figure S2A, Supporting Information), demonstrating that PDCC culture generated an extremely high content of cancer cells. Somatic single nucleotide variants (SNVs) analysis showed that PDCCs maintained a majority of CRC‐associated somatic mutations detected in parental tumors (**Figure** **3**A). The mean allele frequency of mutations found in PDCCs (0.49% ± 0.14%) was higher than that found in the corresponding parental tumors (0.24% ± 0.13%, *p* < 2.22e‐16; Figures S2B,C and S3, Supporting Information). PDCCs maintained 90.2% of the SNVs detected in parental tumors (Figure 3B; Figure S2D,E, Supporting Information). Moreover, most tumor/PDCC pairs displayed similar patterns of copy number alterations (CNAs) (Figure 3C). Furthermore, the comparison of genomic alterations from early and late passage PDCCs indicated that both SNVs and CNVs were well maintained even after long‐term culture in vitro (*n* = 2; Figure 3D; Figure S2F,G, Supporting Information). Overall, 2D PDCCs faithfully maintain the genomic landscapes present in their parental tumors.

### 2.4 Transcriptomic Analysis of 2D PDCCs and 3D ALI Organotypic Cultures

**Figure 4:**
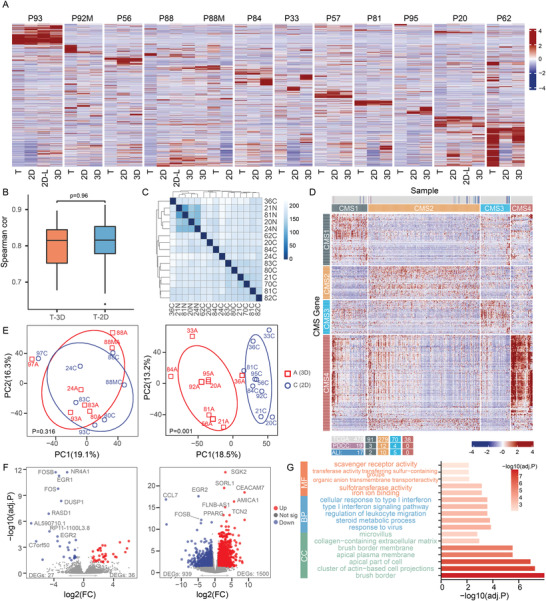
Transcriptome profiles of PDCCs, ALI organotypic cultures and matched primary tumors. A) Heatmap of 2000 highly variable genes between PDCCs (2D), ALI organotypic cultures (3D), and tumor tissues (T) in RNA‐seq expression data. 2D‐L denotes long‐term cultured 2D PDCCs. P93: after 10 passages; P88: after 10 passages; P20: after 20 passages. *n* = 12, different patient samples. B) Spearman correlation based on overall gene expression level between tumor tissues and ALI organotypic cultures (T‐3D; cor = 0.79 ± 0.06), as well as between tumor tissues and PDCCs (T‐2D; cor = 0.8 ± 0.07). Value represents the mean ± SD. *n* = 12, different patient samples. The *p*‐value is determined by PERMANOVA test. No significant difference between them (*p* = 0.96). C) Correlation heatmap of PDCCs from 11 different patients and four NSCs. Samples are color coded by distance. C) PDCCs; N, NSCs. D) The consensus molecular subtypes in PDCCs and ALI organotypic cultures. RNA‐seq data of 19 PDCC lines, 17 ALI organotypic cultures are normalized and combined with 478 TCGA CRC samples. The distribution of PDCCs and ALI organotypic cultures among TCGA are indicated by colored lines. E) PCA shows that seven matched 2D and 3D pairs were clustered together (left, *p* = 0.316), while the other nine matched 2D and 3D pairs were divided into two distinct clusters (right, *p* = 0.001). The significance was determined by PERMANOVA test. 2D: PDCCs; 3D, ALI organotypic cultures. F) Volcano plot of DEGs in 2D PDCCs (blue) versus 3D ALI organotypic cultures (red). Only 63 DEGs are present in the 2D and 3D pairs with similar gene expression profiles (left), while 2439 DEGs are present in the 2D and 3D pairs with significantly different gene expression profiles (right). Adjusted *p*‐value (<0.05) and log2 fold‐change (absolute value >1) are used to select DEGs. G) Bar plots of the enriched GO terms of upregulated genes in 3D ALI organotypic cultures derived from 2D and 3D pairs with significantly different gene expression profiles.

To evaluate whether 2D PDCCs and 3D ALI organotypic cultures represent the transcriptomic landscape of the primary tumors from which they were derived, we performed RNA sequencing (RNA‐seq) on patient‐matched 2D PDCCs, 3D ALI organotypic cultures, and parental tumors from 12 individuals, as well as NSCs from four patients. Here, both 2D PDCCs and 3D ALI organotypic cultures had a high correlation with their corresponding parental tumors in gene expression (the spearman correlation coefficient was 0.8 ± 0.07 and 0.79 ± 0.06, respectively; **Figure** **4**A; Figure S4A, Supporting Information). Importantly, the 2D and 3D models showed no significant difference in the correlation with their parental tumors (*p* = 0.96; Figure 4B). Consistent with a previous study,^[^ ^11c^ ^]^ the correlation heat map showed that NSCs were clustered together, while the PDCCs were clustered separately from the NSCs and exhibited much more heterogeneity (Figure 4C). Finally, RNA‐seq data also showed stable gene expression profiles of long‐term cultured PDCCs from three patients (Figure 4A; P93: after 10 passages, P88: after 10 passages; P20: after 20 passages).

To further characterize these PDCCs and ALI organotypic cultures, we combined their expression data with 478 CRC tissue expression data from the 2012 Cancer Genome Atlas (TCGA), and identified their subtypes using consensus molecular CRC classifier (Figure 4D).^[^ ^17^ ^]^ Except for three of the 17 patients, PDCCs and ALI organotypic cultures derived from the same individuals displayed the same subtype. These PDCC and ALI organotypic cultures samples represented most of the subtypes, CMS2 subtype was the most common, followed by the CMS3 subtype. No PDCC was classified as the CMS4 subtype that enriched expression of genes related to epithelial to mesenchymal transition or stromal infiltration, consistent with the patient‐derived organoids model.^[^ ^18^ ^]^ This may be due to the lack of stromal cells from tumor tissues in PDCCs.

We were curious to know whether the gene expression profiles between 2D PDCCs and 3D ALI organotypic cultures were very similar or significantly different. Interestingly, the analysis indicated that both conditions existed (Figure 4E,F). Among the 16 patient‐matched samples, seven matched 2D and 3D pairs were very similar in gene expression profiles, with only 63 differentially expressed genes (DEGs), and without any enriched signaling pathways. However, nine matched 2D and 3D pairs were significantly different in gene expression profiles, with up to 2439 DEGs. Gene ontology (GO) enrichment analysis showed that DEGs between 3D ALI organotypic cultures and PDCCs were mainly involved in signaling pathways related to differentiation and extracellular matrix, which were overlapped when compared with NSC and their ALI structure (Figure 4G; Figure S4B,C, Supporting Information). It should be noted that even though there were 1000–3000 DEGs between 2D and 3D, this was still a small proportion of the total number of genes detected and some DEGs may be correlated to parental tissue at different expression levels, which may explain why 2D and 3D were equally correlated with the primary tumor in the gene expression profile. In addition, we also compared transcriptome data of PDCCs with their corresponding spheroids, and they were very close in gene expression (Figure S4D, Supporting Information).

In summary, both 2D PDCCs and 3D ALI organotypic cultures maintain the transcriptomic profile of the corresponding primary tumors, and both range across the CRC molecular subtypes. The differences in gene expression between 2D and 3D models ranged from small to large. The reason for this divergence may be due to the different differentiation potential between PDCCs. Whether this difference in gene expression between the 2D and the 3D models will lead to differences in their drug response will be answered in the following section.

### 2.5 Patient‐Specific Drug Sensitivities of 2D PDCCs and 3D ALI Organotypic Cultures

To evaluate this 2D and 3D culture system as a functional model for drug testing, we performed proof‐of‐concept drug response in both 2D PDCCs and 3D ALI organotypic cultures. First, we subjected 20 pooled PDCC lines from 20 patients to chemotherapeutic agents 5‐fluorouracil (5‐FU), oxaliplatin and irinotecan, as well as to seven targeted agents including nutlin‐3a (a stabilizer of TP53), Trametinib (MEK 1/2 inhibitor), and Akt Inhibitor VIII (an AKT inhibitor), Panobinostat (HDAC inhibitors), gefitinib (EGFR inhibitor), PLX4720 (BRAF inhibitor), and AZD8931(EGFR, ErbB2 and ErbB3 inhibitor). PDCCs were quantitatively dispensed into 10% Matrigel‐coated 96‐well plates, and cell viability assay was performed 6 d after drug treatment. Each agent was conducted with five concentrations, three technical replicates and two biological replicates per PDCC line. The half‐maximal inhibitory concentration (IC_50_) and dose–response curves were used to assess the PDCCs drug sensitivity.^[^ ^11^, ^19^ ^]^ The IC_50_ values between technical and biological replicates were highly correlated (Pearson correlation Rp of 0.91 and 0.82, respectively; Figure S5A, Supporting Information).

**Figure 5:**
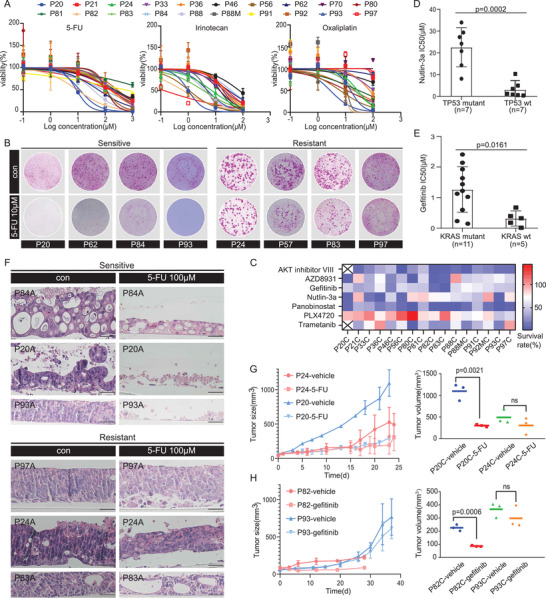
The 2D and 3D model captures intertumoral heterogeneity of drug response. A) Dose–response curves after 6 days treatment of 20 PDCC lines with 5‐fluorouracil (5‐FU), oxaliplatin, and irinotecan generated from luminescence signals. *n* = 3, technical replicates. Value represents the mean ± SD. B) The functional phenotypes of drug response indicated by 2D PDCC colonies treated with 10 × 10^−6^ m 5‐FU. Four PDCC lines are sensitive, four PDCC lines are resistant. Cells were fixed, Rhodamine stained, and photographed after six days treatment. Three technical replicates for each PDCC line. C) Heatmap of dose response of seven drugs in PDCC lines (*n* = 16). The mean survival from triplicate experiments is displayed. D) Scatter plots of IC_50_ (µm) values after six days of treatment of PDCCs with nutlin‐3a in association with TP53 mutation status. All experiments are carried out in triplicate, and data are represented as means ± SD. Mutant PDCCs (circle, *n* = 7), wild‐type PDCCs (square, *n* = 7); *p* = 0.0002. The *p*‐value is calculated by two‐tailed Student's *t*‐test. E) Association of KRAS status and gefitinib response. All experiments are carried out in triplicate, and data are represented as means ± SD. Mutant PDCCs (circle, *n* = 11), wild‐type PDCCs (square, *n* = 5); *p* = 0.0161. The *p*‐value is calculated by two‐tailed Student's *t*‐test. F) H&E staining of ALI organotypic cultures derived from three sensitive PDCC lines show obviously damaged after treatment with 100 × 10^−6^ m 5‐FU for six days, while ALI organotypic cultures derived from three resistant PDCC lines show small changes. Three technical replicates are performed for each PDCC line, *n* = 6. Scale bar: 50 µm. G) In vivo activity of 5‐FU in xenografted tumors derived from PDCCs of P20 and P24 (nude mice, *n* = 3 per group). Mice were injected subcutaneously with PDCCs and treated when tumor volumes reached 50 mm^3^. A significant difference in tumor size between the 5‐FU and the vehicle treatment was observed in P20 (sensitive PDCCs), but no difference in P24 (resistant PDCCs). Results are shown as the tumor volume (mean ± SD). Tumor volumes are determined by measuring at the last time point (*p* = 0.0021; right). The *p*‐values are calculated by two‐tailed Student's *t*‐test. H) In vivo activity of gefitinib in xenografted tumors derived from PDCCs of P82 and P93 (nude mice, *n* = 3 per group). There was no difference between gefitinib and vehicle treatment in P93 (resistant PDCCs), while the growth rate of P82 PDCC (sensitive) derived xenografts treated with gefitinib was much slower than the control group (*n* = 3 per group). Results shown as tumor volume (mean ± SD). Tumor volumes are determined by measuring at the last time point (*p* = 0.0006; right). The *p*‐values are calculated by two‐tailed Student's *t*‐test.

Pooled PDCC lines exhibited striking differences in response to the chemotherapeutic agents (**Figure** **5**A). In order to validate our screening method, we further performed a 5‐FU sensitivity test on 2D PDCC colony cultures, and the results of the two assays were completely consistent (Figure 5B). Only a few PDCC lines were in drug sensitive groups that exquisitely sensitive to 5‐FU (P20, P62, P84, P93), oxaliplatin (P20, P56, P82, P93), and irinotecan (P20, P56, P82).

PDCCs displayed heterogeneous responses to targeted agents (Figure 5C; Figure S5B, Supporting Information). In most cases, the variations in drug response were attributed to particular somatic mutations. For instance, seven PDCC lines harboring wild‐type TP53 (IC50 = 0.37 × 10^−6^ –10.32 × 10^−6^ m) were more sensitive to Nutlin‐3a than those harboring mutant TP53 (IC50 = 8.07 × 10^−6^ –33.99 × 10^−6^ m; Figure 5D). However, some variations in drug response were inconsistent with the expected somatic mutations, which highlight the value and advantage of functional drug‐sensitivity testing in vitro using CRC PDCCs. For example, we confirmed a positive association between KRAS mutations and gefitinib resistance (*p* = 0.0161), but there were still exceptions (Figure 5E). Of the 16 patients tested, only P56, P82, and P93 carried EGFR mutations. Expectedly, the KRAS wild type P82 PDCCs were most sensitive to gefitinib (IC50 = 0.01424 × 10^−6^ m), and the KRAS mutant P93 PDCCs were resistant (IC50 = 1.025 × 10^−6^ m). However, the KRAS wild type P56 PDCCs showed resistance to gefitinib (IC50 = 1.606 × 10^−6^ m). The underlying mechanism for this discordance should be further investigated.

We were curious to know if the 2D drug responses were consistent with their corresponding 3D cultures, since we already demonstrated varying degrees of difference in gene expression profiles between 2D and 3D models. For this, we compared the drug response between 2D PDCCs and 3D ALI organotypic cultures. Once ALI organotypic cultures formed stable 3D structures, 100 × 10^−6^ m 5‐FU was added to the ALI organotypic cultures for six days. Then, the drug sensitivity was characterized by histological analysis. First, four ALI organotypic cultures were generated from four PDCC lines that responded differently to 5‐FU. Interestingly, the 5‐FU response of the 3D ALI organotypic cultures was highly consistent with that of the 2D PDCCs (Figure 5F). Interestingly, the PDCCs derived from P84 and P20 that showed significant difference in gene expression profile with their ALI organotypic cultures were sensitive to 5‐FU, while the PDCCs derived from P83, P97, P24 that showed little difference in gene expression profile with their ALI organotypic cultures were resistant to 5‐FU. Regardless of the difference in gene expression profiles, the drug response results showed a high concordance between 2D PDCCs and 3D ALI organotypic cultures. The results also indicated that tumor cells with relatively high differentiation potential were more sensitive to chemotherapy drugs, while poorly differentiated cells were more resistant.

Finally, the PDCC drug response in vitro was validated in a mouse xenograft model. Four representative PDCC lines with different responses to 5‐FU or gefitinib were subcutaneously injected into mice. Significant growth inhibition was observed in drug‐treated xenografts originating from sensitive PDCCs (P20 and P82), while continued growth was observed in vehicle or drug‐treated xenografts originating from resistant PDCCs (P24 and P93) (Figure 5G,H). These results suggest that drug sensitivity of PDCCs in vitro is consistent with those in vivo.

In conclusion, these findings indicate that both 2D PDCCs and 3D ALI organotypic cultures can reveal intertumoral heterogeneity of drug response. This 2D and 3D model has proved to be a good functional model for drug testing.

### 2.6 The 2D PDCCs Reveal Intratumoral Heterogeneity in Drug Response

Cancer cells within each cancer patient have been found to be extensively heterogeneous in response to treatment. Moreover, treatment can also have a significant impact on tumor cellular composition, drug resistant subclones that pre‐exist at the time of diagnosis usually dominate at the time of recurrence.^[^ ^20^ ^]^ Therefore, it is necessary to develop models that can study the heterogeneity within tumors in vitro, which can test the efficacy of new targeted treatments and new strategies for customizing treatment combinations for individual tumors. The 2D model showed extremely high proliferation rate and high efficiency when generating single‐cell‐derived clones from normal stem cells, so we wonder whether the optimized model can be used to culture single cells derived from individual tumors for studying the intratumoral heterogeneity.

**Figure 6:**
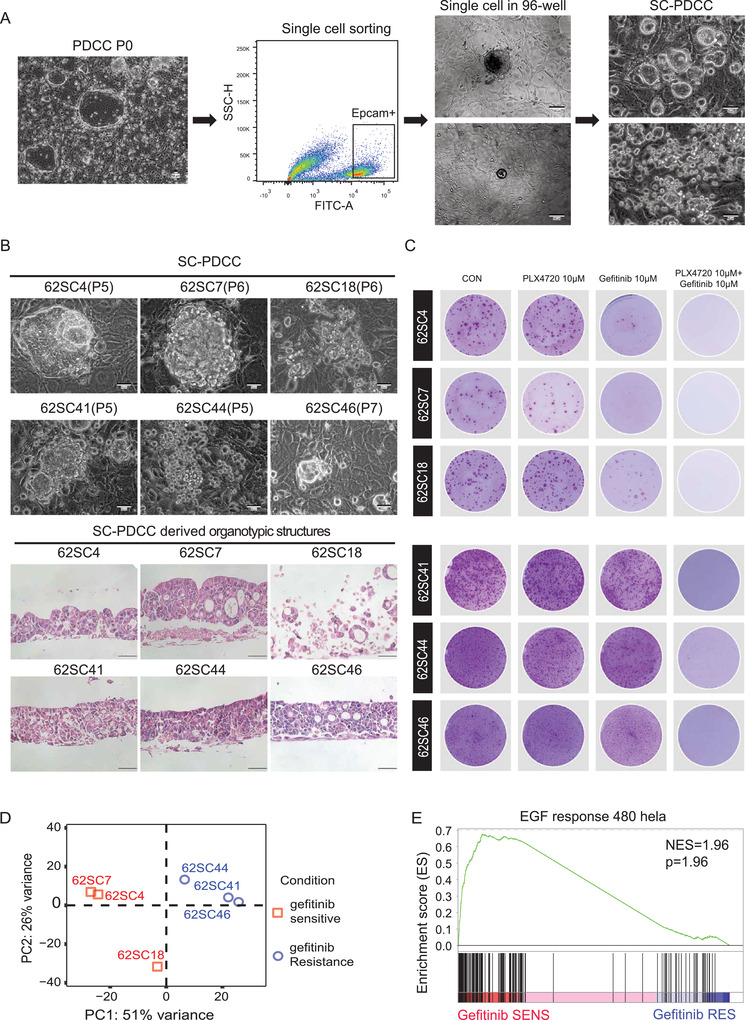
SC‐PDCC clones capture intratumoral heterogeneity of drug response. A) Schematic procedure of establishing SC‐PDCC clones from CRC patient. Colonies were generated after processing tissues (PDCC P0). Scale bar: 50 µm. Then cells were sorted using EPCAM^+^ to obtain single cell per well. Scale bar: 100 µm. SC‐PDCC clones were established after expansion. Scale bar: 50 µm. B) Representative bright field microscopy images of SC‐PDCC colonies and H&E images of SC‐PDCCs derived ALI organotypic cultures. Passage numbers were marked in brackets. Scale bar: 50 µm. C) Rhodamine staining showing that all SC‐PDCC clones of P62 are resistant to 10 × 10^−6^ m PLX4720, but their responses to gefitinib are different. All PLX4720 resistant SC‐PDCC clones are sensitive to the combination of 10 × 10^−6^ m PLX4720 and 10 × 10^−6^ m gefitinib. Three technical replicates are performed for each clone. D) PCA showed that SC‐PDCC clones with different response to gefitinib are separated into two groups. E) GSEA showing the significantly enriched EGF response pathway in gefitinib sensitive SC‐PDCC clones.

We generated the first pooled PDCCs after digesting the primary tumor cells on the feeder cells. Then, we sorted the Epcam^+^ single cell to the 96‐well plates precoated with feeder cells for cell expansion. The success rate of generating SC‐PDCC clones is 42–57% (*n* = 576; **Figure** **6**A). The SC‐PDCCs derived from P62 showed different morphological characteristics (Figure 6B). The ALI organotypic cultures derived from SC‐PDCCs also displayed different histologic characteristics (Figure 6B).

We tested the intratumoral drug response of SC‐PDCCs derived from P62 by EGFR inhibitor gefitinib. And we found some SC‐PDCCs were sensitive to gefitinib while others were resistance (Figure 6C). We analyzed the differentially expressed genes between them and found that relatively sensitive SC‐PDCCs were characterized by the intrinsic activation of EGF signaling (Figure 6D,E).

PLX4720 is a selective inhibitor of BRAF V600E with proven therapeutic efficacy in melanoma models harboring BRAF V600E.^[^ ^21^ ^]^ We found that P62 was harboring activating BRAF V600E mutations. Therefore, we tested whether PLX4720 was effective to SC‐PDCCs derived from P62. However, the results showed that all SC‐PDCCs had a very limited response to this drug. The drug resistance mechanism may be due to the feedback activation of EGFR caused by PLX4720 inhibition of BRAF(V600E), as previously reported.^[^ ^22^ ^]^ We therefore adopted the strategy of combining PLX4720 and gefitinib to sensitize those clones that were resistant to both, and the result showed a marked inhibition of proliferation (Figure 6C). We provided evidence that colon cancer patients harboring the BRAF(V600E) oncogenic mutation were lack a significant response to PLX4720, but BRAF and EGFR inhibition can be synergistic when combined, suggesting more combining targeted agents can be used in clinical trials.

Overall, we can successfully establish single‐cell‐derived clones in the early generation of primary tumor cell cultivation to preserve the intratumoral heterogeneity, which lays a good foundation for finding pre‐existing drug‐resistant clones and exploring their drug‐resistant mechanism in the future. Thus, our data suggest that 2D model could serve as an effective platform to study the intratumoral heterogeneity of drug response and guide drug treatment against colorectal cancer.

### 2.7 The SC‐PDCCs Reveal Intratumoral Heterogeneity of Tumorigenicity

There is a hierarchy of tumorigenic and nontumorigenic cells in tumors. For example, according to the theory of “cancer stem cell”, only minority cell populations are tumorigenic, and the most other cells have little contribution to tumor growth.^[^ ^23^ ^]^ It is very important to identify the tumorigenic cells and treat them. So, it will be crucial to continue to optimize culture models to capture and characterize these cells that retain the tumorigenic potential to promote tumor growth.

**Figure 7:**
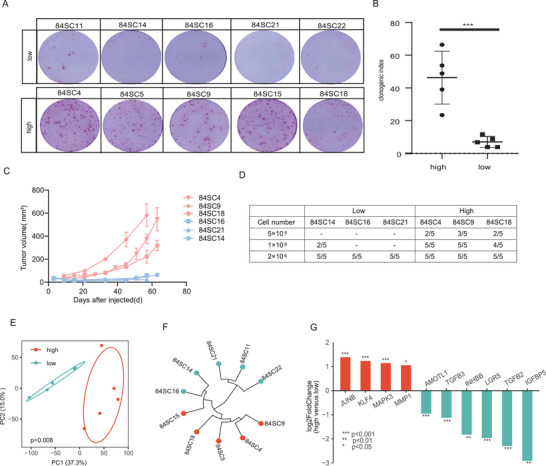
SC‐PDCC clones capture intratumoral heterogeneity of tumorigenicity. A) Rhodamine staining showing the clonogenicity assay of colonies grown 15 days on feeder cells following seeding 1000 single cells per well from each SC‐PDCC clone of P84 patient; *n* = 3, biological replicates. B) Quantification of clonogenicity based on percentage of colony formation; *n* = 3, biological replicates data represented as mean ± SD. *p* < 0.0001. C) The growth curve of the xenograft generated from SC‐PDCC clones with different tumorigenicity (nude mice, *n* = 5 per SC‐PDCC clone). Red represents high tumorigenicity, blue represents low tumorigenicity. D) Table summarizing the number of injected cells of the SC‐PDCC clones in P84, as well as the proportion of xenograft formation. E) PCA showed that SC‐PDCC clones with high or low tumorigenicity were separated into two groups. *p* = 0.008. F) Unsupervised hierarchical clustering was performed on SC‐PDCC clones with high or low tumorigenicity. G) Top upregulated DEGs in SC‐PDCC clones with high tumorigenicity (red) and SC‐PDCC clones with low tumorigenicity (blue). **** adjust *P* < 0.0001, *** adjust *P* < 0.001, ** adjust *P* < 0.01, * adjust *P* < 0.05, based on RNA‐seq data.

We assessed whether SC‐PDCCs from the same individual patient exhibited intratumoral heterogeneity in tumorigenicity. We examined the clonogenic ability in total of ten SC‐PDCC clones from P84. The clonogenicity of SC‐PDCC clones in P84 varied from 7.02% ± 3.34% to 46.27% ± 16.12% (**Figure** **7**A,B). We also carried out further verification through the subcutaneous tumor formation experiment in mice. We tested three concentration gradients 5 × 10^5^, 1 × 10^6^, 2 × 10^6^ for transplantation assays (Figure 7C,D). The results showed that the clones with high clonogenic ability had strong ability to form tumors in vivo. 84SC21, 84SC16, and 84SC18 formed tumors very slowly and the tumor stopped after it grew to a certain volume, but 84SC4, 84SC9, and 84SC18 can form tumors continuously.

Using the strategy of integrating molecular heterogeneity and intrinsically distinct tumorigenicity potential into single cells with the same personalized genetic landscape, we further analyzed tumorigenicity‐associated genes. We identified 918 DEGs between high and low tumorigenic SC‐PDCC clones using RNA‐seq data (*P* ≤ 0.05, |log2 Ratio| ≥ 1; Figure 7E,F). LGR5, TGFB2, IGFBP5, TGFB3, INHBB, AMOTL1, TWIST2, TWIST1 were highly expressed in clones with low tumorigenic ability. The clones with high tumorigenic ability expressed JUNB, KLF4, MAPK3, MMP1, CEACAM6, CEACAM1, MUC17, FABP1, ID1 (Figure 7G). GO functional enrichment analysis indicated that these DEGs were mainly involved in the cancer proliferation, cell cycle in IR response pathway and TGF‐*β* signaling pathway (Table S3, Supporting Information).

We found that stem cell‐related pathways were not enriched in SC‐PDCCs with high tumorigenic ability, which implicates that tumorigenic cancer cells do not necessarily possess the characteristics of stem cells. These results provide evidence that not all tumorigenic colorectal cancer cells follow the cancer stem cell model.^[^ ^23a^ ^]^ In future research, we can screen out some biomarkers that can distinguish clones with different tumorigenic ability, and perform corresponding functional verification to develop therapeutic targets specifically for clones with high tumorigenicity.

## 3 Discussion

We are entering the era of precision medicine, with an increasing reliance on and demand for cancer models. Although cancer cell lines are often described as inadequate models that do not effectively predict human responses,^[^ ^9^, ^24^ ^]^ there is sufficient evidence that large cell line panels (such as NCI60, GDSC, and CCLE)^[^ ^8^, ^25^ ^]^ can capture the genomic diversity of human cancers and meet the needs for in vitro preclinical evaluation.^[^ ^9^, ^10^ ^]^ However, the number of traditional patient‐derived cancer cell lines is limited and their generation efficiency is extremely low. This makes it practically impossible to study intertumoral heterogeneity, and most importantly, intratumoral heterogeneity. In this study, we have efficiently established a large number of stable cell lines from CRC that accurately retain the genomic and transcriptomic landscapes of the corresponding tumors of origin. Here, we provided the first direct comparison and proof that 2D cells and 3D structures display an equal correlation with their parental tumors in terms of gene expression, and displayed the same trend in drug response in vitro. The issue of whether 2D and 3D models have the same correlation with patients’ clinical drug response should be further compared in future studies.

In recent years, 3D organoid culture is a key technological breakthrough in the stem cell field and cancer research developed by Clevers and co‐workers. Multiple cancer types of patient‐derived tumor organoids can be efficiently established, can faithfully recapitulate more physiological features of their parental tumors, and also can undergo long‐term expansion in culture and maintain genome stability.^[^ ^12^, ^26^ ^]^ These standard organoids have a basal‐out structure and a closed “lumen,” making it difficult to access the apical surface.^[^ ^27^ ^]^ Organoid culture is highly dependent on relatively rigid Matrigel, which to some extent hampers drug penetration.^[^ ^28^ ^]^ However, the 3D organotypic cultures generated in ALI system have an open “lumen” that maintain the apical‐basal polarity and of parental tumors. They are uniform in shape, depending on the size of the transwell. In addition, they are multilayered tumor cells that is physiologically closer to the histological features of their parental tumors. In ALI organotypic culture system, the Matrigel was replaced by 3T3‐J2 feeder cells, which is a type of stroma cells used to support the physiological microenvironment.^[^ ^12^ ^]^ Complementarily, our 2D model takes advantage of high cell proliferation rates to provide sufficient cell resources for ALI organotypic culture in a short period of time. Notably, both standard organoids and ALI organotypic cultures lack immune cells, thus hindering immunotherapy assessment and research.

Concerning the difference between 2D and 3D models, under certain conditions cells lose their ability to differentiate in the 2D model, but display their differentiation potential in the 3D model.^[^ ^15^, ^28^ ^]^ We systematically demonstrated the outstanding advantages of 2D models in terms of simple technical operation, high‐throughput screening, and high cell proliferation rate for supply of cell resource in a short time, and the unique advantages of 3D models in terms of histological architecture, multicellular components, and ECM microenvironment. Accordingly, we have demonstrated that the combination of 2D and 3D models can be applied to preclinical research more effectively and systematically, fully compensating for their shortcomings and exploiting their advantages. We should also compare the patient derived organoids directly derived from the original tissues with the corresponding 2D PDCCs to further evaluate their similarities and differences in future research.

Notably, 2D models can also capture the inter‐ and intratumoral heterogeneity of drug sensitivities, which enable to isolate and identify pre‐existing drug resistant subclones from the primary tumors and to explore the mechanism underlying drug resistance. Therefore, we emphasize here that 2D models have been previously unjustly accused of not being able to represent the primary tumor, not because of the 2D culture itself, but because of the extremely low efficiency of generating 2D patient‐derived cancer cell lines. Thereby, our study recalls the value of 2D cancer models in guiding the personalized treatment for cancer patients. In view of these advantages, we need to further explore and verify whether this powerful 2D and 3D model can capture and evaluate the molecular heterogeneity and multiple functional heterogeneity of individual cancer cells in colorectal cancer.

Most importantly, we have shown the intratumoral heterogeneity of tumorigenicity in primary CRC. We found that at the transcriptional level, there are significant DEGs and signaling pathways between clones with distinct tumorigenicity. This resource provides valuable information for exploring the molecular mechanisms underlying the tumorigenic ability of cancer cells. This individual primary CRC we tested had a goblet cell‐like phenotype, and their tumorigenic cells might be considered a minority of “cancer stem cell.” However, the molecular feature of these tumorigenic cells in this individual was completely unrelated to stem cell identity. This founding sheds light on the complexity and heterogeneity of clonal tumorigenicity in primary CRC tissues, and in this case rules out the “cancer stem cell” theory.

## 4 Experimental Section

### Patient Samples

The study was approved by the ethics committee of institution review board of the Tsinghua University (#20170019 and #20190303), National Cancer Center/Cancer Hospital, Chinese Academy of Medical Sciences, and Peking Union Medical College (#19/172‐1956). Normal and CRC tissue samples were obtained from patients who were diagnosed with CRC and underwent surgical resection at the hospital. All patients gave written informed consent. Information on cancer and noncancer tissue specimens was shown in Table S1 of the Supporting Information.

### 2D PDCC Culture

Colorectal tumor tissue was excised after surgery, stored, and transported in wash buffer: F12 (Gibco), 5% FBS (Hyclone), 1% penicillin/streptomycin (Gibco), 0.1% Amphotericin B (Gibco), 0.25% Gentamicin (Gibco), 1% HEPES (Gibco), and 5 × 10^−6^ m Rock inhibitor (Calbiochem) at 4 °C. Complete the follow‐up treatment within 48 h. Tissue samples were divided into three parts. One piece was fixed and sectioned for histology, one piece was used for extraction of DNA and RNA, the remainder used for PDCCs culture. In some cases, however, the amount and quality of the DNA or RNA did not reach the required standard for accurate analysis because of the paucity of tissue specimens.

Tumor tissues were cut into small pieces and incubated in 1 mg mL^−1^ collagenase type XI buffer (Gibco) at 37 °C for 10–15 min. The digested cell solution was filtered through a 70 µm cell strainer (Falcon), and washed four times with wash buffer. Isolated cells were resuspended in SCM culture medium: Advanced DMEM/F12 (Hyclone) supplemented with 10% FBS, 1% penicillin/streptomycin, 1% l‐glutamine (Hyclone), 0.1% Amphotericin B, 0.5% Gentamicin, 0.18 × 10^−3^ m Adenine (Sigma), 5 µg mL^−1^ Insulin (Sigma), 2 × 10^−9^ m T3 (Sigma), 0.2 µg mL^−1^ Hydrocortisone (Sigma), 125 ng mL^−1^ R‐Spondin 1(R&D), 1 × 10^−6^ m Jagged‐1(AnaSpec Inc), 100 ng mL^−1^ Noggin (Peprotech), 2.5 × 10^−6^ m Rock inhibitor (Merck), 2 × 10^−6^ m SB431542 (Cayman chemical), 10 × 10^−3^ m Nicotinamide (Sigma), and 10 ng mL^−1^ EGF (Peprotech). After resuspension, the cells were seeded onto irradiated 3T3‐J2 feeder cells that were paved one day in advance, and cultured at 37 °C in 7.5% CO_2_. The culture medium was replaced every two days. PDCCs were digested in a 0.25% trypsin‐EDTA solution (Gibco) at 37 °C for 5 to 8 min with passage every 7 to 10 days. When the normal morphology was observed in the primary culture plates, colonies were then propagated into the medium without R‐Spondin1 to remove normal ISCs contamination.

PDCCs were resuspended in freezing medium comprised of SCM medium supplemented with 30% FBS and 10% dimethyl sulfoxide (DMSO, Sigma), and placed in cryovials for storage at −80 °C. For recovery, vials were quickly thawed in a 37 °C water bath, and PDCCs were resuspended using fresh SCM medium.

### 3D Air–Liquid Interface Organotypic Culture and 3D Matrigel Spheroids Culture

ALI organotypic culture was performed as described.^[^ ^15^ ^]^ Each Transwell insert (Corning) was coated with 20% Matrigel (growth factor reduced, BD Biosciences) and incubated at 37 °C for 30 min to polymerize. 200 000 feeder cells were seeded into each Transwell insert and incubated overnight at 37 °C in 7.5% CO_2_. PDCCs were digested in a 0.25% trypsin‐EDTA solution at 37 °C for 8 to 10 min and passed through 30 µm filters (Miltenyi Biotec) to obtain single cells. The pellets were resuspended in 80 µL F12 and 20 µL mouse feeder removing beads (Miltenyi Biotec) and incubated at 4 °C for 15 min avoid light. Placed columns in the magnetic MACS Separator and rinsed with 3 mL F12 buffer. Then added 400 µL F12 to the cells before passing through the columns. Finally, purified PDCCs were eluted with another 2 mL F12. 200 000–300 000 PDCCs were seeded into each Transwell insert and cultured with SCM medium. After reaching confluency (3–7 days), the top medium was removed by carefully pipetting, and the PDCCs were further incubated for 6–12 days in SCM minus nicotinamide medium (SCM‐6) before analysis. The SCM‐6 medium was changed every day.

Colorectal cancer spheroids were generated under PDO condition as described previously.^[^ ^11^, ^16^, ^29^ ^]^ PDCCs were digested and purified by removing mouse feeder cells as mentioned above. The pellet was resuspended in organoid culture medium. 1 × 10^5^ cells were embedded in Matrigel and seeded in 24‐well plates (30 µL of Matrigel per well). Following polymerization (20 min, 37 °C), the gels were overlaid with 500 µL of organoid culture medium (Advanced DMEM/F12 containing Glutamax, penicillin–streptomycin, HEPES, 1 × B27 (Gibco), 1 × N2 (Gibco), 1.25 × 10^−3^ m n‐Acetyl Cysteine (Sigma), 10 × 10^−3^ m Nicotinamide, 50 ng mL^−1^ human EGF, 100 ng mL^−1^ human Noggin, 500 ng mL^−1^ R‐spondin1, 10 × 10^−9^ m Gastrin (Sigma), 500 × 10^−9^ m A83‐01(Sigma), 3 × 10^−6^ m SB202190 (Sigma), and 10 × 10^−9^ m Prostaglandine E2 (Santa Cruz Biotechnology). Organoid culture medium was refreshed every two days.

### SC‐PDCC Culture

When the colonies were generated after processing tissue, cells were digested in a 0.25% trypsin‐EDTA solution (Gibco) for 5 to 8 min at 37 °C and cell suspensions were passed through 30 µm filters (Miltenyi Biotec). 10^6^ cells were blocked with 0.1% FBS at 4 °C for 30 min, then incubated with CD326 Monoclonal Antibody (Thermo Fisher Scientific, 53‐9326‐42, 1:50) at 4 °C for 30 min. Samples were collected and sorted with an Aria SORP Cell Sorter (BD). Single cells were seeded into 96‐well plates coated with feeder cells.

### Immunohistochemistry

Sections of formalin‐fixed, paraffin‐embedded tissues, xenografts, and ALI organotypic cultures were stained by standard HE staining. For immunohistochemistry staining, slides were subjected to antigen retrieval in citrate buffer (pH 6.0, Sigma‐Aldrich) at 95 °C for 20 min, and a blocking procedure was performed overnight with 5% bovine serum albumin (Sigma‐Aldrich,) and 0.05% Triton X‐100 (Sigma‐Aldrich) in DPBS(‐) (Gibco) at 4 °C. Primary antibodies used in this study included antibodies against MUC2 (Santa Cruz Biotechnology, SC‐15334, 1:500), KI67 (Thermo Fisher Scientific, 550609, 1:1000). Then, sections were stained using DAB Substrate Kit (#550880, BD). Images were acquired by Olympus IX73 microscopy.

### Cell Viability Assay

PDCCs were trypsinized into single cells and 5000 cells were seeded into 96‐well plates coated with 10% Matrigel, in triplicate. Cells were cultured in SCM medium for 0, 24, 48, and 72 h, and measured cell viability using with CellTiter‐Glo reagent (Promega).

### Drug Screening

Stock solutions of all drugs were prepared at 10 × 10^−3^ m in DMSO, including chemotherapeutic agents 5‐fluorouracil (5‐FU), oxaliplatin, and irinotecan as well as targeted agents nutlin‐3a (a stabilizer of TP53), trametinib (MEK 1/2 inhibitor), Akt Inhibitor VIII, Panobinostat (HDAC inhibitor), gefitinib (EGFR inhibitor), PLX4720 (BRAF inhibitor), and AZD8931 (an EGFR, ErbB2 and ErbB3 inhibitor). All drugs were purchased from Selleck. PDCCs were gently disrupted into single cells and feeder cells were removed with magnetic beads as mentioned above. 96‐well plates were coated with 10% Matrigel. 10 000 cells per well were plated in triplicate in 96‐well plates and allowed to adhere for 24 h before treatment with drugs at increasing concentrations or with DMSO as a negative control. MG‐132 at 4 × 10^−6^ m and staurosporin at 2 × 10^−6^ m were used as positive controls. After 6 days, 100 µL CellTiter‐Glo (Promega) was added to each well. The plates were agitated for 30 min at RT prior to luminescence reading. Each drug was performed with three technical replicates and two biological replicates with different passages. The results were normalized to controls and expressed as percent cell viability. The determination of IC_50_ values was conducted using Graph Pad Prism9. After obtaining IC_50_, PDCC lines to each drug were divided into three subgroups based on their IC_50_ values: sensitive group (lowest 33.3%), moderate response group (middle 33.3%), and resistant group (top 33.3%).^[^ ^30^ ^]^

### 2D PDCCs and 3D ALI Organotypic Cultures Drug Responsiveness Test

For 2D PDCCs, the drugs were added to SCM medium at final concentration of 10 × 10^−6^ m, and added on the second day of PDCCs culture. 0.1% DMSO was added to SCM as control. Following six days of treatment, the cells were washed twice with PBS and fixed with 4% paraformaldehyde for 20 min, then stained with 10% rhodamine staining solution. Surviving cells were counted under a bright‐field microscope. PDCCs with survival rate greater than 50% were defined as drug‐resistant, and PDCCs with survival rate less than 50% were defined as drug‐sensitive.

For 3D ALI organotypic cultures, the drugs were added to SCM‐6 medium after confluency (5–7 days). After six days of treatment, the ALI organotypic cultures were washed twice with PBS, formalin‐fixed, and embedded in paraffin for HE staining.

### Subcutaneous Injection

Mice aged 6 to 12 weeks were maintained under pathogen‐free conditions, and in accordance with Tsinghua University Animal Ethics Committee guidelines. PDCCs were harvested and subcutaneously injected into BALB/c Nude mice at 10^6^ cells in 100 µL 50% Matrigel. When the mean tumor size reached between 50 and 100 mm^3^, the mice were randomly treated with either vehicle, Gefitinib (80 mg kg^−1^, orally, 5 days on, 2 days off), or 5‐FU for 4 weeks. Gefitinib was freshly prepared in corn oil before gavage administration. 5‐FU was intraperitoneally injected three times a week at the dose of 25 mg kg^−1^. Animals were sacrificed with CO_2_ before combined tumors reached a volume of 1200 mm^3^. Tumor size was determined every three days by caliper measurements, and tumor volume (in mm^3^) was calculated by using the following formula: *v* = 0.5 × length × width^2^.

### Determination of Cell Clonogenic Ability

PDCCs growing in logarithmic phase were gently disrupted into single cells and feeder cells were removed with magnetic beads as mentioned above. 1000 cells were planted on 12 well plates after counting. Shake the plates gently and replace the fresh medium every two days. Medium was removed after 15 days of growth. Washed twice with PBS and fixed them with 4% paraformaldehyde. Then, stained them with 10% rhodamine for 20 min, wash them with water, dry them after excess staining, and count the number of cell clones formed.

### Whole‐Exome Sequencing (WES) Analysis

Genomic DNA was extracted with DNeasy Blood & Tissue kit (Qiagen). 1–3 µg DNA was used for WES. Single base mutations, insertions, and deletions were identified by first aligning the Illumina pair‐end reads to the mouse reference genome (GRCm38), and using Bowtie2 (version 2.3.2) to remove the contamination DNA of mouse cells from feeder layer. Then, filtered reads were quality‐filtered by trim galore, aligned to human reference genome (hg19) by BWA (version 0.7.15) [Aligning sequence reads, clone sequences, and assembly countings with BWA‐MEM] and transfer to BAM files by Samtools (version 1.3.1) respectively.^[^ ^31^ ^]^ Next, Picard (version 2.18.27) was used to mark duplicate reads from BAM files, and Genome Analysis Toolkits (version 4.1.0.0) was used to recalibrate with default parameters.

Somatic mutations in tumor samples were called by comparison to their matched adjacent normal tissues using Mutect2. The parameters 1) –af‐of‐alleles‐not‐in‐resource was set to 0.0000025 to filter germline variant, and 2) –annotation was set as UniqueAltReadCount. Subsequently, GATK FilterMutectCalls (–unique‐alt‐read‐count 5) and FilterByOrientationBias were used to perform second filtration with default parameters. Variants were annotated to the functional consequence using ANNOVAR (version Apr 2018) based on human genome build hg19.^[^ ^32^ ^]^ CNAs were called using FACETS (version 0.5.14), which are integer copy number calls that correct for tumor purity, ploidy, and clonal heterogeneity.^[^ ^33^ ^]^ Tumor purity and ploidy were extracted from the resultant for downstream analysis.

### RNA‐seq Analysis

RNA was isolated from PDCCs and tissues with Trizol Reagent (Invitrogen) and with RNeasy mini kit (QIAGEN). 2 µg of prepared input RNA was sequenced on the Illumina platform generating 150 bp paired‐end reads. RNA‐seq reads were mapped and quantified using HISAT2 (version 2.0.5)‐StringTi (version 1.3.4) pipeline.^[^ ^34^, ^35^, ^36^ ^]^ Differential expression analysis for high abundance genes (mean reads > 10) was performed by DESeq2 (version 1.24.0).^[^ ^37^ ^]^ Adjusted p value (<0.05) and log_2_ fold‐change (absolute value > 1) was used to select significantly DEGs for further downstream pathway analysis. Gene set enrichment analysis (GSEA) and gene ontology term enrichment analysis (GOEA) were performed with clusterProfiler (version 3.12.0). ^[^ ^38^ ^]^ GOEA was carried out based on DEGs with an adjusted *p*‐value less than 0.01.

The variation coefficient (CV) of genes among different samples derived from the same individual were calculated. Next, high abundance genes (mean of read > 50) were selected for downstream analysis. The principal components analysis (PCA) was performed based on the top 10 000 CV genes by the function prcomp of the package ggbiplot (version 0.55) in R. PERMANOVA was carried out using Adonis from the Vegan (version 2.5.6) R package to identify significance between different groups.

### The Correlation between PDCCs and Tissue Sample

To assess the overlap of somatic nonsynonymous variants (SNVs) in tissues and matched PDCCs, all sample‐specific mutations were researched among the unfiltered mutations in the compared samples to avoid missing mutations occurring in very few cells. SNVs (Frame_Shift_Del, Frame_Shift_Ins, Splice_Site, Translation_Start_Site, Nonsense_Mutation, Nonstop_Mutation, In_Frame_Del, In_Frame_Ins, and Missense_Mutation) located in CRC driver genes were visualized with oncoplot by R package Maftools (version 2.0.16) among 23 paired samples.^[^ ^39^ ^]^

To assess the gene expression characteristics of the PDCCs and corresponding parental cancer tissues, a Z‐score based method was used to normalize two groups. For each group, the TPM normalized expression data for each gene was scaled to Z‐score among sample. Then, the two groups of scaled expression data were combined and screened using 2042 genes with CV (>1) and mean of TPM (>1) and plotted with the function of heatmap (clustering_method_columns = “ward.D2”) from R package ComplexHeatmap (version 2.0.0).^[^ ^40^ ^]^ The Spearman and Pearson correlation coefficient were calculated in the R function cor (). The corresponding *p*‐value of the correlation coefficient was calculated in R function cor.test ().

### Subtype Sample

Our 19 PDCC lines, 17 ALI organotypic cultures and 478 samples from TCGA‐COAD were selected for this analysis. Transcriptome data were used to classify samples into CMS group by R package CMSclassifier with the “single‐sample predictor” algorithm.^[^ ^17^ ^]^ The advantage of this algorithm is that the results are constant, whether it is predicted individually or over a series of samples. To maximize the ability to analyze results, findings and conclusions were presented using the “nearest‐CMS” classification.

### Quantification and Statistical Analysis

Analysis procedures of genome, transcriptome data were provided in the relevant sections of Method Details Statistical analysis was performed with GraphPad Prism and presented as mean values ± SD. Two‐tailed Student's *t*‐test was used to calculate *p*‐values between two groups. Corresponding statistical significance was denoted with (∗ *p* < 0.05; ∗∗ *p* < 0.01; ∗∗∗ *p* < 0.001, and ∗∗∗∗ *p* < 0.0001) in the figures and figures legends.

## Conflict of Interest

The authors declare no conflict of interest.

## Authorship Contributions

Y.Z. and B.Z. contributed equally to this work. Y.Z. generated and cultured all of the PDCCs and ALI organotypic cultures performed most of the biological experiments and data analysis, prepared figures, figure legends, and methods. B.Z. analyzed all sequencing data and prepared methods. Y.M. generated 3D spheroids. F.Z. and J.C. provided patient specimens and maintained clinical records. B.W. performed histopathological analysis. H.J, F.Z., J.G., and Q.Z. participated additional data collection and analyses. H.W. supervised tumor tissue collection and generation of spheroids. Q.L. supervised tumor tissue collection and clinical records. F.Z. supervised sequencing data analysis. X.W. supervised project design and wrote manuscript.

## Supporting information

Supporting Information

## Data Availability

The data that support the findings of this study are openly available in https://ngdc.cncb.ac.cn/, reference number PRJCA006448.
