# Supplementary material for: Colorectal Cancer Patient‐Derived 2D and 3D Models Efficiently Recapitulate Inter‐ and Intratumoral Heterogeneity
Source: Adv Sci (Weinh). 2022 Jun 2;9(22):2201539. doi: 10.1002/advs.202201539 (PMC9353492; doi:10.1002/advs.202201539)
Supplement: Supplementary file 1 — Supporting Information [file ADVS-9-2201539-s001.pdf]

ADVANCED SCIENCE
Open Access

Supporting Information

for *Adv. Sci.*, DOI 10.1002/advs.202201539

Colorectal Cancer Patient-Derived 2D and 3D Models Efficiently Recapitulate Inter- and Intratumoral Heterogeneity

Yuanyuan Zhao, Bing Zhang, Yiming Ma, Fuqiang Zhao, Jianan Chen, Bingzhi Wang, Hua Jin, Fulai Zhou, Jiawei Guan, Qian Zhao, Hongying Wang, Qian Liu, Fangqing Zhao* and Xia Wang**

Supporting Information

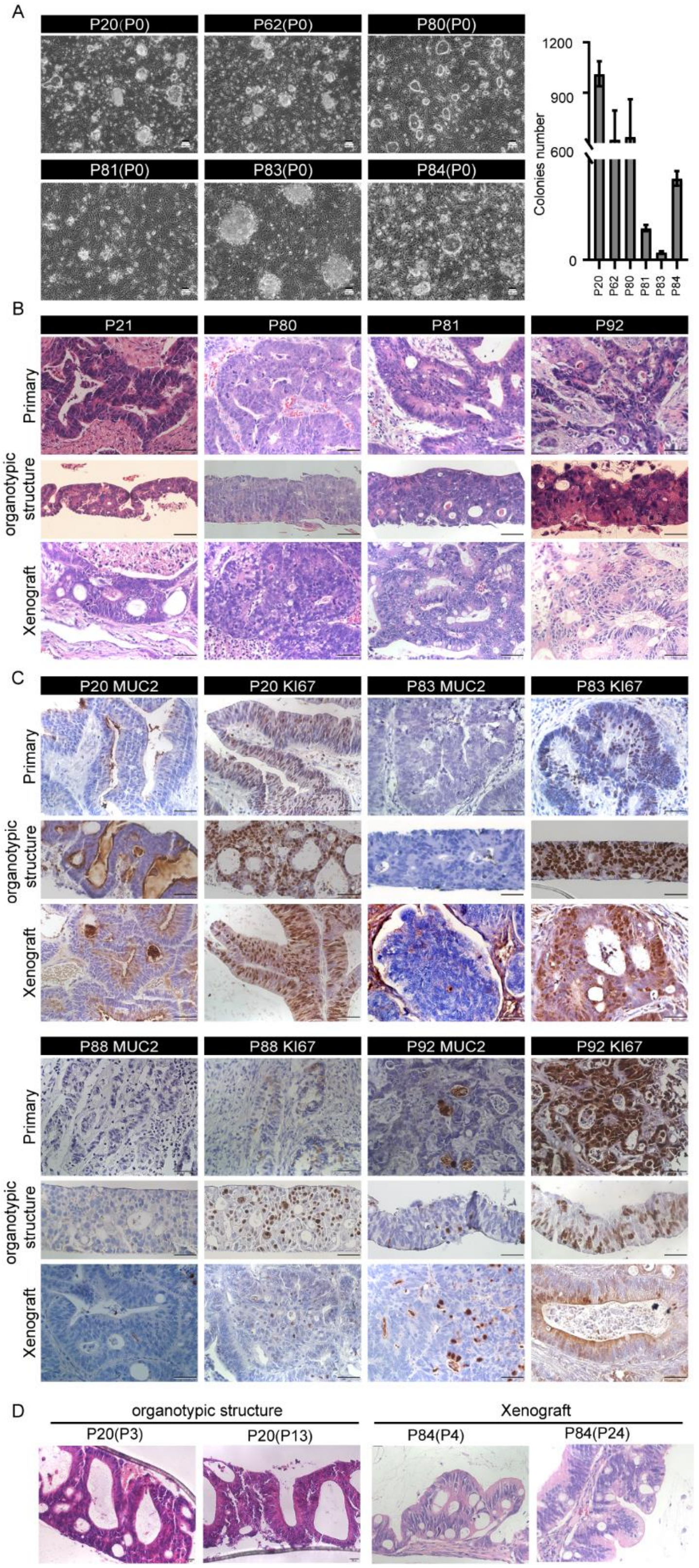

Figure S1. 3D ALI organotypic cultures and Xenografts Retain Histologic Features of their Parental Tumors, related to Figure 1.

(A) Brightfield images of PDCC colonies directly derived from colorectal cancer tissues, passage numbers are P0. P0: first culture of the primary tumors. Scale bar, 100 μm. The statistics of the number of colonies are listed on the right.

(B) H&E staining images of four additional representative parental tumors, corresponding 3D ALI organotypic cultures and xenografts. Scale bars, 50 μm.

(C) MUC2 and KI67 IHC staining of representative patient matched parental tumors, ALI organotypic cultures, and xenografts. Scale bar, 50 μm.

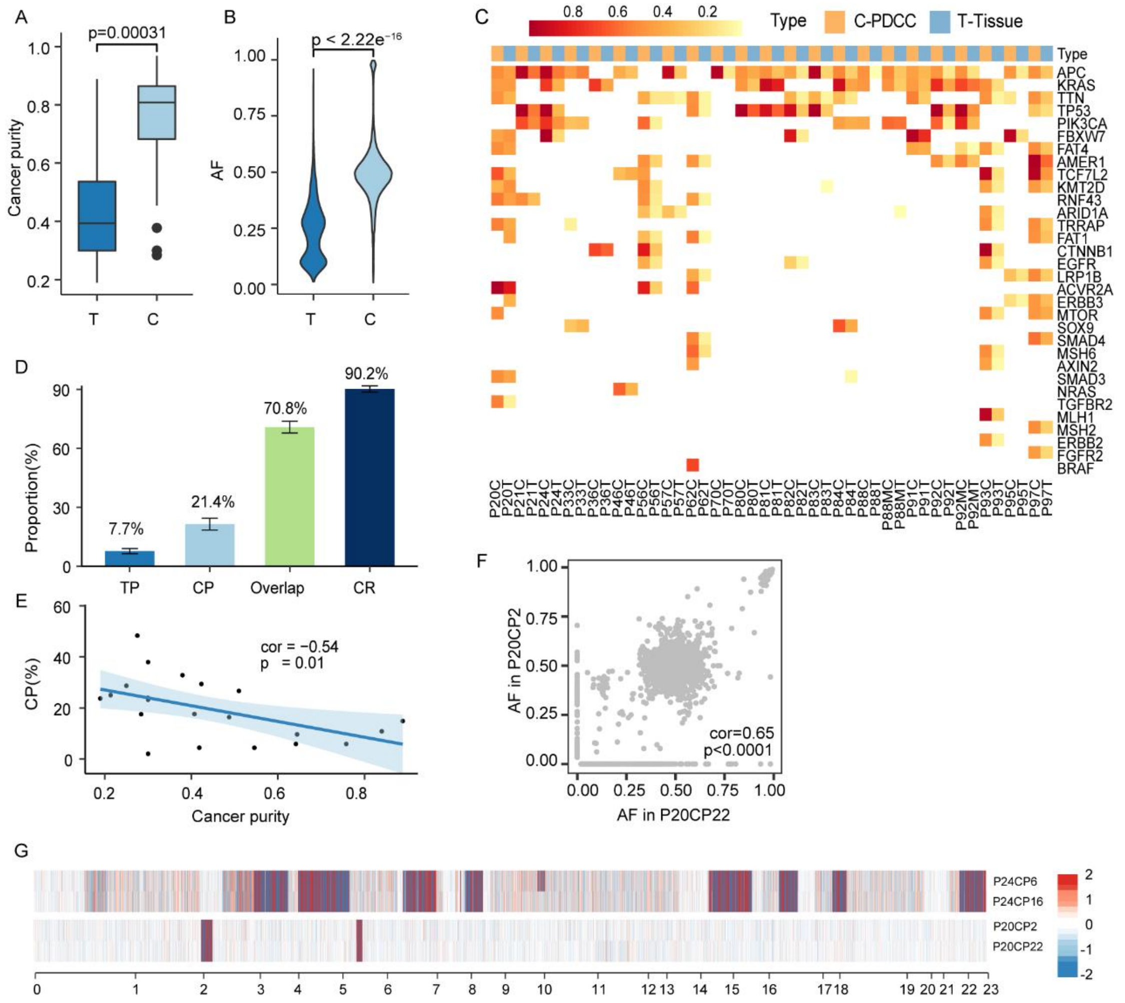

Figure S2. PDCCs Recapitulate Genomic Alterations of Parental Tumors, related to Figure 3.

(A) Box-whisker plots showing higher tumor purity in PDCCs than tumor tissues (n = 23, different patient samples; p = 0.00031, Wilcox test).

(B) Violin plots showing higher allele frequencies in PDCCs than that in tumor tissues. (n = 23, different patient samples; p < 2.22e-16, Wilcox test).

(C) The allele frequencies for driver mutations in PDCCs and corresponding tumor tissues.

(D) Proportion of shared and unique mutations between PDCCs and tissues. The mutation proportion only present in tumor tissues is 7.7% (private SNV of tissue, TP), only present in PDCCs is 21.4% (private SNVs of PDCCs, CP), the overlap in PDCCs and tissues is 70.8%, and the 90.2% mutations detected in tissues are present in the PDCCs (retained SNVs in PDCCs, CR).

(E) The number of private SNVs of PDCCs (CP) is negatively correlated with the tumor purity (Cor = - 0.54; p = 0.01). Correlations and p values are calculated by Pearson's correlation method.

(F) Scatterplot comparing the somatic mutant fraction of SNVs for short-time (passage 2) and long-time culture (passage 22) of P20. Cor = 0.65; p < 0.0001. Correlations and p values are calculated by Pearson's correlation method.

(G) Heatmap showing CNAs in short-term cultured PDCCs (passage 6) and long-term cultured PDCCs (passage 16) of P24. Pearson correlation coefficients is 0.96.

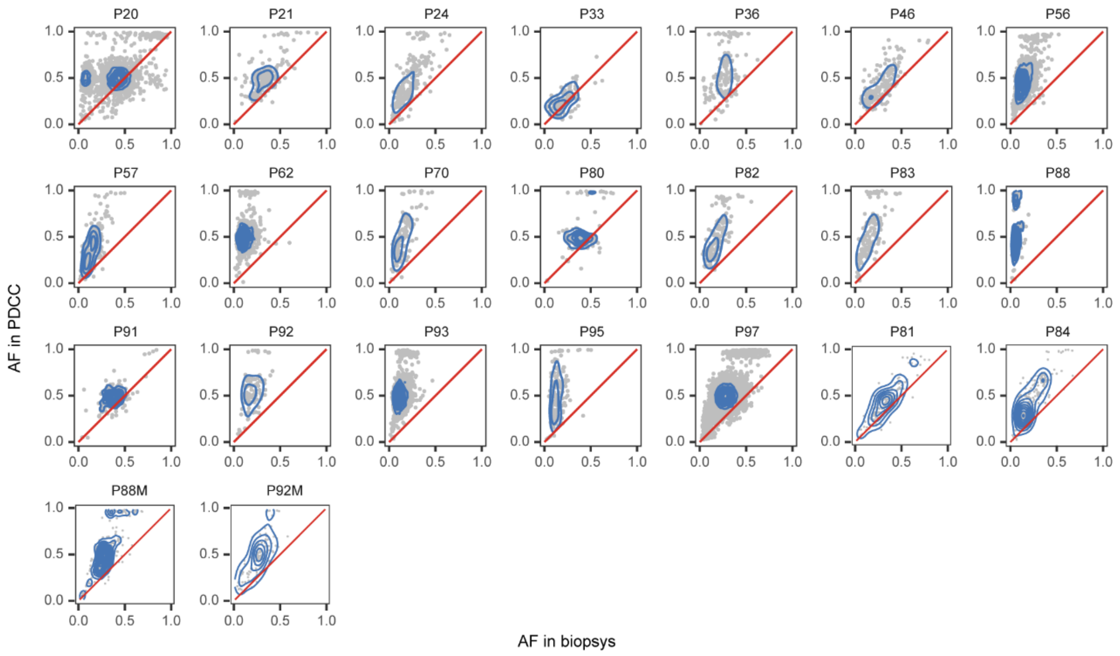

Figure S3. The correlation of allele frequencies in PDCCs and corresponding tumor tissues, related to Figure 3.

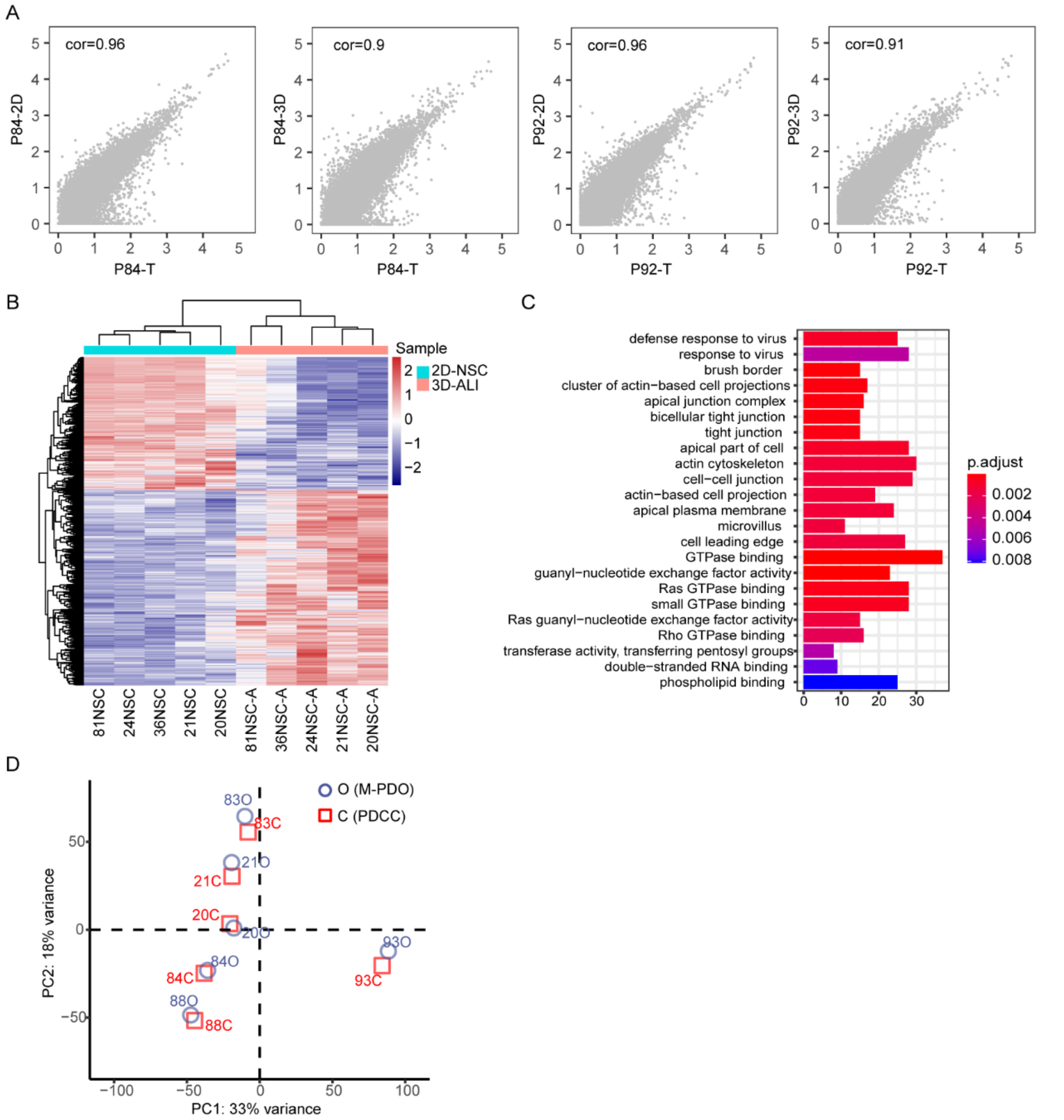

Figure S4. Transcriptome characteristics in PDCCs and Normal Stem Cells, related to Figure 4.

(A) Representative scatterplot illustrating the transcriptome correlation between PDCCs and their corresponding tumors (2D vs T), as well as between ALI organotypic cultures and their corresponding tumors (3D vs T). Correlation coefficient was marked in the figure, $p < 0.0001$. Correlations and p values are calculated by Pearson's correlation method.

(B) Heatmap showing the difference between the NSCs and their corresponding ALI

organotypic cultures.

(C) Bar plots of the enriched GO terms of upregulated genes in 3D ALI organotypic cultures compared with 2D NSCs.

(D) PCA shows that PDCCs and their derived spheroids are very close in gene expression. C, PDCCs; O, PDCC derived spheroids.

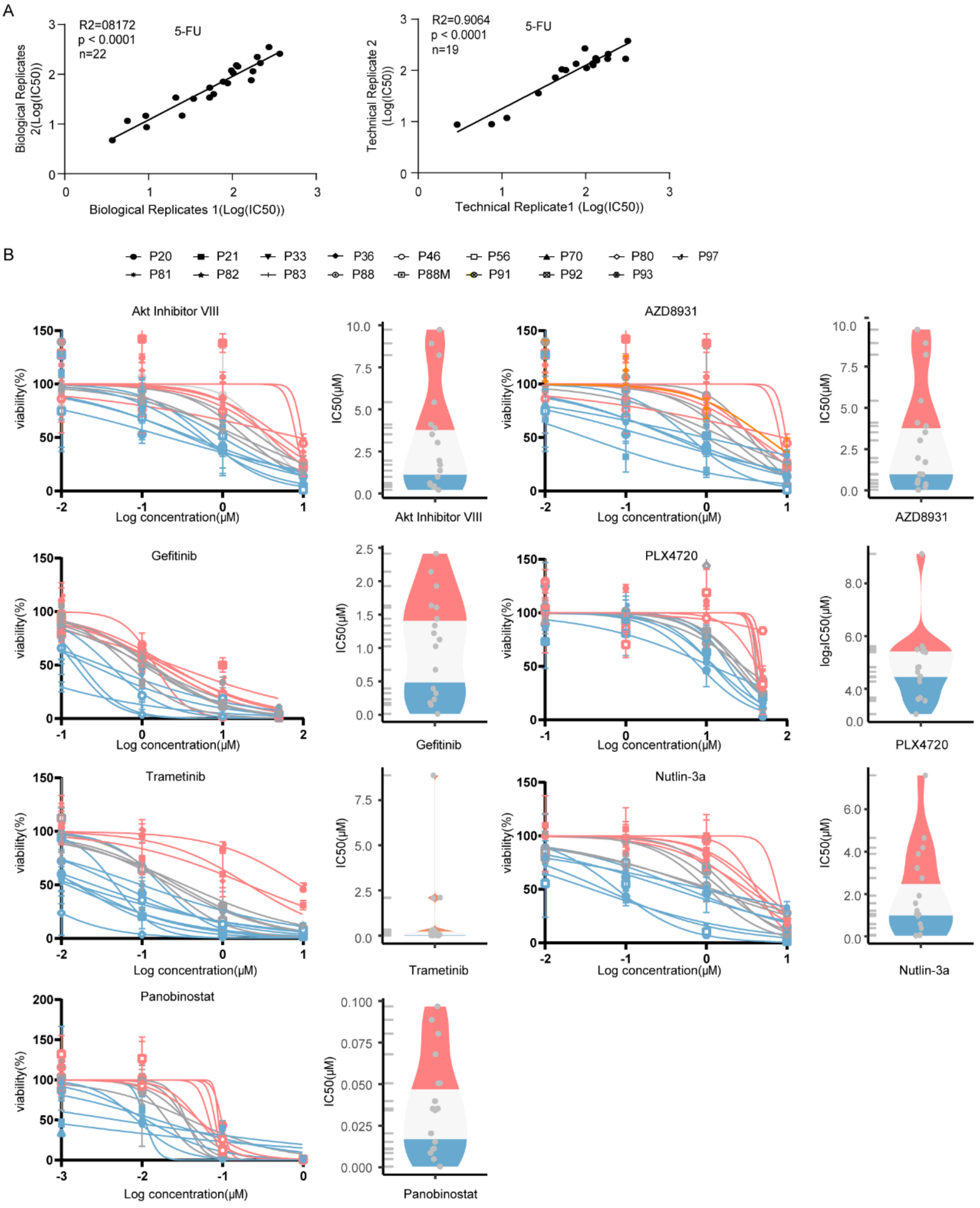

Figure S5. Drug Response of PDCCs, related to Figure 5.

(A) Reproducibility of drug response profiles for 5-FU. Plots show correlation between the two biological replicates for each PDCC line using different passages (left, $n = 22$, $p < 0.0001$). Each screen was conducted two times of technical replication (right, $n = 19$, $p < 0.0001$). Each data point represents $\log(IC_{50})$. The p-values are calculated by two-tailed Student's t-test.

(B) Dose-response curves of PDCC after six days treatment with seven targeted drugs, respectively. Error bars represent SD of three independent experiments. Violin plots show the distribution of IC_{50} values of the drugs in the 17 PDCC lines except PLX4720 which is indicated by $\log_2(IC_{50})$. The blue portion represents the 33% most-sensitive samples, the red portion the 33% most-resistant samples, and the middle portion intermediate drug responses.

Supplementary Information Table 1, Clinicopathological data for 39 colon cancer patients, related to Figure 1.

**Patient sample**	**Source**	**Sex**	**Age**	**Pre-treatment**	**Stage**	**metastases**	**Histology grade**	**Normal counterpart**	**2D-PDCC culture**	**ALI-PDO culture**	**M-PDO culture**	**Subcutaneous tumo**	**WES**	**PDCC RNA-Seq**	**ALI-PDO RNA-Seq**	**Subtype Biopsy**	**Subtype 2D PDCC**	**Subtype ALI-PDO**	**Passage ratio**
P20	E	M	47	N	III	-	mod/poorly	+	+	+	+	+	+	+	+	Goblet.like	Goblet like	Goblet like	1:200
P21	E	M	66	N	III	-	well/mod diff	+	+	+	+	+	+	+	+		TA like	TA like	1:50
P24	E	M	66	Y	IV	-	mod diff	+	+	+	N/T	+	+	+	+		TA like	TA like	1:100
P33	E	M	49	N	III	-	mod diff	+	+	+	N/T	+	+	+	+	Enterocyte	Stem like	Goblet like	1:4
P34	E	F	84	N	II	-	mod diff	N/T	-										
P35	E	M	67	N	III	-	mod diff	N/T	-										
P36	E	F	67	N	IV	-	mod/poorly	+	+	+	N/T	+	+	+	+	Goblet.like	Goblet like	Goblet like	1:4
P44	E	M	55	N	IV	+	poorly diff	N/T	-										
P45	E	F	63	N	IIIb	+	poorly diff	N/T	-										
P46	E	M	43	N	III	-	mod diff	+	+	N/T	N/T	N/T	+	+	N/T	TA	TA like		1:10
P53	E	M	76	N	IV	-	mod/poorly	+	+	N/T	N/T	N/T	N/T	N/T	N/T	Enterocyte			1:6
P54	E	F	74	N	IV	-	mod/poorly	N/T	+	N/T	N/T	N/T	N/T	N/T	N/T				1:6
P56	E	M	65	N	III	-	mod diff	+	+	+	N/T	N/T	+	+	+	Enterocyte	TA like	TA like	1:10
P57	E	M	60	Y	IV	+	mod/poorly	N/T	+	N/T	N/T	+	+	+	N/T	Enterocyte	TA like	TA like	1:6
P61	E	M	82	N	II	-	mod diff	N/T	-										
P62	E	F	64	N	III	-	mod diff	+	+	+	+	+	+	+	+	Goblet.like	Goblet like	Goblet like	1:100
P70	E	M	62	N	II	-	mod/poorly	+	+	+	N/T	+	+	+	N/T	Enterocyte	TA like		1:6
P71	E	M	70	N	IV	-	mod/poorly	N/T	-										
P74	E	M	75	Y	III	-	mod/poorly	N/T	+	N/T	N/T	N/T	N/T	N/T	N/T				
P76	E	M	57	N	III	-	mod/poorly	N/T	+	N/T	N/T	N/T	N/T	N/T	N/T				
P80	E	M	62	N	III	-	mod/poorly	+	+	+	N/T	+	+	+	+		TA like	TA like	1:6
P81	E	F	47	N	II	-	mod/poorly	+	+	+	N/T	+	+	+	+	Goblet.like	TA like	TA like	1:7
P82	E	M	72	N	III	-	mod diff	+	+	+	N/T	Not fo	+	+	N/T		TA like		1:7
P83	E	M	78	Y	IV	+	poorly diff	+	+	+	+	+	+	+	+		TA like	TA like	1:10
P84	E	M	66	N	III	-	mod diff	+	+	+	+	+	+	+	+	Goblet.like	Goblet like	Goblet like	1:10
P86	E	M	70	Y	II	+	mod/poorly	N/T	-										
P87	E	M	65	N	III	+	mod/poorly	N/T	+	N/T	N/T	N/T	N/T	N/T	N/T				
P88	E	M	61	N	IV	+	mod/poorly	+	+	+	+	+	+	+	+	Enterocyte	TA like	Goblet like	1:20
P88M	E	M	61	N	IV	+	mod/poorly difff	+	+	+	N/T	+	+	+	+	Inflammato	Goblet like	Goblet like	1:50
P90	E	M	70	Y	II	-	mod diff	N/T	+	N/T	N/T	N/T	N/T	N/T	N/T				

P91	E	M	79	Y	III	-	mod diff	+	+	+	N/T	Not fo	+	+	N/T	Goblet.like	Goblet like		1:10
P92	E	M	75	N	IV	+	mod diff	+	+	+	+	+	+	+	N/T		TA like		1:6
P92M	E	M	75	N	IV	+	mod diff	+	+	+	N/T	N/T	+	+	+	TA	TA like	TA like	1:10
P93	E	F	51	Y	IV	+	poorly diff	+	+	+	N/T	+	+	+	+	Enterocyte	TA like	TA like	1:40
P94	E	M	66	Y	III	+	mod/poorly	N/T	-										
P95	E	M	71	Y	III	+	mod/poorly	N/T	+	+	N/T	N/T	+	+	+	Enterocyte	TA like	TA like	1:20
P96	E	M	58	Y	I	-	mod/poorly	N/T	+	N/T	N/T	N/T	N/T	N/T	N/T				
P97	E	M	51	Y	IV	-	poorly diff	+	+	+		+	+	+	+		TA like	TA like	1:100
P98	E	F	66	Y	III	+	poorly diff	N/T	-										

E: Surgical excision

N/T: Not Test

Supplementary Information Table 2, Tumor formation in mice, related to Figure 1

PDCCs	Cell injected	engraftment	xenograft	days reached to 500mm
P20	10^6	subcutaneous injection	5/5	19
P21	10^6	subcutaneous injection	5/5	22
P24	10^6	subcutaneous injection	5/5	21
P33	10^6	subcutaneous injection	5/5	>90
P36	10^6	subcutaneous injection	3/6	>90
P57	10^6	subcutaneous injection	5/5	69
P62	10^6	subcutaneous injection	5/5	22
P70	10^6	subcutaneous injection	5/5	36
P80	10^6	subcutaneous injection	5/5	>93
P83	10^6	subcutaneous injection	5/5	>53
P84	10^6	subcutaneous injection	5/5	47
P88M	10^6	subcutaneous injection	5/5	15
P92	10^6	subcutaneous injection	5/5	74
P93	10^6	subcutaneous injection	5/5	40
P97	10^6	subcutaneous injection	5/5	19

Supplementary Information Table 3, Geno Ontology (GO) functional enrichment analysis

ID	Description	GeneRatio	BgRatio	p.adjust	qvalue
hsa04010	MAPK signaling pathway	11/95	294/8102	0.020147	0.016121
hsa04014	Ras signaling pathway	10/95	232/8102	0.020147	0.016121
hsa04015	Rap1 signaling pathway	10/95	210/8102	0.0134	0.010723
hsa04060	Cytokine-cytokine receptor interaction	10/95	295/8102	0.042346	0.033885
hsa04218	Cellular senescence	8/95	156/8102	0.020147	0.016121
hsa04350	TGF-beta signaling pathway	10/187	94/8102	0.015107	0.014408
hsa04915	Estrogen signaling pathway	7/95	138/8102	0.026701	0.021366
hsa05142	Chagas disease	7/95	102/8102	0.0134	0.010723
hsa05146	Amoebiasis	6/95	102/8102	0.026701	0.021366
hsa05205	Proteoglycans in cancer	9/95	205/8102	0.020147	0.016121
hsa05219	Bladder cancer	6/187	41/8102	0.042592	0.04062
hsa05224	Breast cancer	7/95	147/8102	0.032874	0.026306
hsa05226	Gastric cancer	9/95	149/8102	0.01277	0.010218
